# Extraction of Artificial Teeth and Metallic Plate from the Œsophagus after Eighteen Days' Impaction

**Published:** 1871-02

**Authors:** 


*Extraction of Artificial Teeth and Metallic Plate from the Œsophagus after Eighteen days' Impaction.*—The following interesting case occurred in England, and is described by the *Lancet* as follows:

Emma N——, aged twenty-five, whilst in bed on Christmas-eve, dreamed that she was swallowing something hard, and on waking discovered that three artificial teeth fixed to a plate of silver and platinum had disappeared from her mouth. She experienced no difficulty at all in breathing. She could not swallow any solid food, the attempt to do so producing a feeling of sickness; but beef-tea, milk, and broth she found no difficulty in swallowing.

Tne patient was brought into the operating theatre on the 11th inst. Mr. Walton passed a pair of long forceps without meeting with any obstruction. He then passed an œsophagus bougie for the purpose of exploring. The instrument entered the stomach, but grated against something in its passage down the tube. The bougie on being withdrawn showed scratches caused by some foreign body. Judging from the upper—i. e., the last—part of the scratch, the object must have been in the œsophagus, at a point opposite the insertion into the sternum of the third costal cartilages. The scratch itself measured 9 in. in length, and there was abont the same distance from the upper part of the scratch to the point of the bougie which touched the patient's lips during the exploration. Mr. Walton then passed a hooked probang, and caught hold of something that appeared to have a sharp edge. He then withdrew the object slowly and carefully to avoid lacerating the œsophagus. When however, it had been brought to the upper part of the pharynx, the patient made a sudden gulp, and swallowed it again. Mr. Walton then seized a pair of long, curved forceps, and, introducing them into the œsophagus, grasped the artificial teeth, and recovered both them and the plate. No loss of blood ensued, and there was only a slight reddish tint in the mucus which the patient spat out.

Although the metallic plate had been out of order for a long time previous to the accident, the patient had never taken the precaution of removing it at night. It held three teeth on one half of its irregular convex edge. Its entire length was 1⅝ in., and the greatest breath 1 in.

As the operation produced pain, and there must have been more or less tearing of the œsophagœl surface, Mr. Walton deemed it advisable that the patient should, for a time, take no food by the mouth. For the first four days she therefore received enemata of six ounces of beef-tea; for three days more these were increased to eight ounces, and administered every five hours. They were all retained. She then began to take liquid food, and shortly afterwards was placed upon full diet. She left the hospital quite well, and without any appreciable loss of strength or flesh.

